# RNA-seq analysis reveals significant transcriptome changes in huntingtin-null human neuroblastoma cells

**DOI:** 10.1186/s12920-021-01022-w

**Published:** 2021-07-02

**Authors:** Johanna Bensalel, Hongyuan Xu, Michael L. Lu, Enrico Capobianco, Jianning Wei

**Affiliations:** 1 Department of Biomedical Science, Charles E. Schmidt College of Medicine, Florida Atlantic University, Boca Raton, FL 33431 USA; 2 Institute of Data Science and Computing, University of Miami, Miami, FL 33146 USA

**Keywords:** RNA-seq, Transcriptome, Huntingtin, Huntington’s disease, Knock out, CRISPR-Cas9

## Abstract

**Background:**

Huntingtin (Htt) protein is the product of the gene mutated in Huntington’s disease (HD), a fatal, autosomal dominant, neurodegenerative disorder. Normal Htt is essential for early embryogenesis and the development of the central nervous system. However, the role of Htt in adult tissues is less defined. Following the recent promising clinical trial in which both normal and mutant Htt mRNA were knocked down in HD patients, there is an urgent need to fully understand the molecular consequences of knocking out/down Htt in adult tissues. Htt has been identified as an important transcriptional regulator. Unbiased investigations of transcriptome changes with RNA-sequencing (RNA-Seq) have been done in multiple cell types in HD, further confirming that transcriptional dysregulation is a central pathogenic mechanism in HD. However, there is lack of direct understanding of the transcriptional regulation by normal Htt.

**Methods:**

To investigate the transcriptional role of normal Htt, we first knocked out Htt in the human neuroblastoma SH-SY5Y cell line using the CRISPR (clustered regularly interspaced short palindromic repeats)-Cas9 (CRISPR-associated protein 9) gene editing approach. We then performed RNA-seq analysis on Htt-null and wild type SH-SY5Y cells to probe the global transcriptome changes induced by Htt deletion.

**Results:**

In general, Htt has a widespread effect on gene transcription. Functional analysis of the differentially expressed genes (DEGs) using various bioinformatic tools revealed irregularities in pathways related to cell communication and signaling, and more specifically those related to neuron development, neurotransmission and synaptic signaling. We further examined the transcription factors that may regulate these DEGs. Consistent with the disrupted pathways associated with cellular development, we showed that Htt-null cells exhibited slower cell proliferation than wild type cells. We finally validated some of the top DEGS with quantitative RT-PCR.

**Conclusions:**

The widespread transcriptome changes in Htt-null cells could be directly caused by the loss of Htt-mediated transcriptional regulation or due to the secondary consequences of disruption in the gene regulatory network. Our study therefore provides valuable information about key genes associated with Htt-mediated transcription and improves our understanding of the molecular mechanisms underlying the cellular functions of normal and mutant Htt.

**Supplementary Information:**

The online version contains supplementary material available at 10.1186/s12920-021-01022-w.

## Background

Huntingtin protein (Htt) is ubiquitously expressed throughout the body and was identified as the product of the gene mutated in Huntington’s disease (HD), a devastating, autosomal dominant, neurodegenerative disorder with no cure yet available [1]. In the effort to find a cure for HD, most studies have focused on understanding the molecular mechanisms underlying the deleterious effects of mutant Htt in various cellular and animal models. In contrast, direct investigations of the molecular function of normal Htt, especially in the adult tissue, are limited. There is growing consensus that understanding the molecular functions of normal Htt can provide important clues as to how mutant Htt contributes to neurodegeneration and HD pathogenesis. Furthermore, following a recent promising clinical trial with anti-sense oligonucleotides (ASOs) that target both normal and mutant Htt mRNA [2], understanding the role of normal Htt in the adult central nervous system (CNS) has become an even more critical area of investigation.

Htt protein is essential for early embryonic development as demonstrated by the compelling evidence showing that mice homozygous for *huntingtin* gene (*Hdh*) disruption displayed embryonic lethality at ~ 8.5 days of gastrulation [3–5]. The role of normal Htt in adult tissues is less defined due to the lethal effect of knocking out Htt in animals. Although ubiquitously expressed, Htt has the highest expression levels in the brain and testes [6, 7]. In the brain, the expression of Htt is generally higher in neurons than in glia cells [7]. Htt conditional knockout mice that lost Htt expression around embryonic day 15 (E15) or postnatal day 5 exhibited progressive neurodegenerative motor phenotypes and reduced lifespans as adult mice, suggesting that normal Htt is required for the normal function of the CNS [8]. Knockout Htt expression in mouse testes also caused male sterility [8]. Conflicting behavioral consequences of knocking out normal Htt in adult mice have been reported. Dietrich et al., globally inactivated the mouse *Hdh* gene using the tamoxifen-inducible Cre-LoxP system at 3, 6 and 9 months of age and showed that elimination of mouse *Hdh* gene consistently led to progressive motor and behavioral decline, reduced life-span and extensive neuropathology [9]. On the other hand, using inducible Htt knockout mice, Wang et al., reported that Htt depletion in mice older than 4 months did not lead to motor dysfunctions or animal death, however, when Htt was ubiquitously knocked out in 2-month-old mice, the animals eventually died of acute pancreatitis [10]. Six-month partial knockout of Htt in the adult Rhesus striatum (~ 45% reduction in Htt expression) did not cause behavioral or neuropathological changes for 6 months after Htt reduction [11].

Despite the ambiguity of the role of Htt in the adult brain from behavioral studies, cellular studies strongly support that Htt is involved in diverse cellular processes that are important for mature neuronal functions, including vesicular trafficking [12], transcriptional regulations [13, 14], synaptic functions [15, 16] and cellular stress responses [17, 18]. Among these cellular functions, transcriptional regulations have been extensively investigated and most studies have focused on the transcriptional dysregulation in HD. Htt has been shown to interact with numerous transcription factors and regulate a number of gene transcriptions [19]. RNA-sequencing (RNA-Seq) provides unbiased information on transcriptome composition and abundance under different conditions. RNA-seq analysis has been done in multiple cell types in HD, including cortical neurons derived from HD induced pluripotent stem cells (iPSCs) [20], and on brain and blood samples from HD patients [21–24]. These studies revealed mutant Htt-induced transcriptomic changes. However, there is a lack of direct investigation on the transcriptional regulation by normal Htt.

In this study, we knocked out Htt in the human neuroblastoma SH-SY5Y cell line using the CRISPR (clustered regularly interspaced short palindromic repeats)-Cas9 (CRISPR-associated protein 9) gene editing approach. We demonstrated that knocking out Htt has a widespread effect on gene transcription using RNA-seq analysis. Functional analysis of the differentially expressed genes (DEGs) using gene ontology (GO) analysis revealed that the up- and down-regulated DEGs are commonly associated with multicellular organism development and processes, especially with neuron development, consistent with the essential role of normal Htt in early embryogenesis and CNS development. We also identified pathways that are highly enriched in the up- and down-regulated DEGs, respectively, with various bioinformatic tools. Additionally, we examined the transcription factors that may regulate these DEGs. Consistent with our analysis, we demonstrated that HttKO cells had decreased cell proliferation. These results provide valuable information about key genes associated with Htt-mediated transcription and improve our understanding of the molecular mechanisms underlying the cellular functions of normal and mutant Htt.

## Methods

### Cell culture

The human neuroblastoma SH-SY5Y cell line was obtained from Sigma-Aldrich (Cat. No. 94030304) and cultured at 37 °C, 5% CO_2_ in a humidified incubator in Dulbecco's Modified Eagle Medium/F-12 K (DMEM/F12K) medium supplemented with 10% fetal bovine serum (FBS) and 1% antibiotic–antimycotic solution. HEK 293 cells were obtained from American Type Culture Collection (CRL-1573) and cultured at 37 °C in DMEM medium supplemented with 10% FBS and 1% antibiotic–antimycotic.

### Generation of SH-SY5Y HTT knockout cell lines by CRISPR-Cas9

Four guidance RNA (gRNA) sequences targeting the human *huntingtin* gene (*HTT*, Gene ID 3064) with minimal off target effects were designed using the online Synthego CRISPR design tool (https://design.synthego.com/#). The four gRNAs are: gRNA1: HTT + 3116206-ACAAGCCUGAAAGGCAGCUU; gRNA2: HTT + 3116154-GCUGCUCACCCUGAGGUAUU; gRNA3: HTT + 3116100-AGGCUUACUCGUUCCUGUCG and gRNA4: HTT-3116085-GACAGGAACGAGUAAGCCUG. gRNAs were dissolved in nuclease-free Tris–EDTA (TE) buffer at a final concertation of 30 μM. *Streptococcus pyogenes* Cas9 protein with two nuclear localization signals (Cas9 2NLS) was obtained from Synthego and used at a final concentration of 20 μM. sgRNA and Cas9 2NLS at a molar ratio of 9:1 were used to form ribonucleoprotein complex at room temperature, which was then delivered to SH-SY5Y cells using the Neon electroporation system (1100 V, 50 ms width, 1 pulse). Forty-eight hours after electroporation, aliquots of cells were first analyzed for cleavage efficiency by mismatch cleavage assay as described below. Based on the calculated cleavage efficiency, cells transfected with gRNA2 were further serial-diluted to obtain single cell colony in 96-well plates. After colony expansion, colonies were first screened for Htt expression levels by Dot Blot (Additional file 1: Figure S1) and positive clones were selected for further validation by Sanger sequencing.

### Mismatch cleavage assay

Forty-eight hours after electroporation, ~ 2 × 10^5^ cells were pelleted and resuspended in 50 μl QuickExtract™ DNA extraction solution (Epicentre). Genomic DNA was extracted by incubating the samples at 65 °C for 10 min followed by inactivation at 95 °C for 5 min. The resulting samples were further diluted twice with water. Mismatch cleavage assay was performed with GeneArt® genomic cleavage detection kit (Invitrogen). Briefly, standard PCR was performed to amplify the Htt fragment containing the mutations with AmpliTaq Gold® 360 DNA polymerase. The following primers were used: 5′- GTAGGTCAGTTGACAGTTTCTCCT-3′ and 5′- TCGTGACCTCTGGTTTTAGAAACA-3′. After PCR amplification and confirmation with DNA gel electrophoresis, PCR products were denatured and re-annealed to form heteroduplex according to the manufacturer’s instruction. The heteroduplex was then incubated with the detection enzyme at 37 °C for 1 h. The cleaved product was immediately analyzed by DNA gel electrophoresis.

### Sanger sequencing and KO analysis

Genomic DNA from stable clones were extracted using QuickExtract™ DNA extraction solution as described above. PCR was performed using the high fidelity Q5 DNA polymerase (NEB) with the following primers: 5′- GTAGGTCAGTTGACAGTTTCTCCT-3′ and 5′- TCGTGACCTCTGGTTTTAGAAACA-3′. A single DNA band at 792 bp was confirmed by DNA gel electrophoresis. PCR product was further column-purified (Qiagen) and sent for Sanger sequencing (GENEWIZ). The forward sequencing primer used was: 5′-CTCTGGAAAGGACCTTGCTGAG-3′. The obtained Sanger sequencing files were uploaded to online CRISPR analysis software, Inference of CRISPR Edits (ICE) from Synthego (http://ice.synthego.com), to determine the KO efficiency and type of edits.

### Immunostaining and image analysis

Cells grown on No. 1.5 coverslips coated with poly-L-lysine were fixed in 4% paraformaldehyde (PFA) in phosphate-buffered saline (PBS) for 15 min at 37 °C. Cells were then permeabilized with 0.25% Triton X-100 in PBS for 10 min. After fixation and permeabilization, cells were immunostained as we described previously [25, 26]. Rabbit monoclonal anti-huntingtin (1:500, D7F7, Cat #5656S, Cell Signaling) was used. Immunofluorescence was detected using a laser confocal microscope (Nikon A1R). DAPI (4′,6-diamidino-2-phenylindole) was used as a nuclear counterstain. Images were taken with a 60X oil objective (CFI Plan Apochromat Lambda 60X Oil, numerical aperture (NA) = 1.4) under a Nikon A1R laser scanning confocal microscope with a Galvano scanner. DAPI signals were imaged using a 405 nm diode laser at 1% power and detected with a photomultiplier tube (PMT). Htt signals were imaged using a 514 nm diode laser at 2.6% power and detected with a GaAsP detector. NIS-elements acquisition software was used to acquire the images with a resolution of 1024 × 1024 pixels.

A Nikon Eclipse Ts2R microscope equipped with a DS-Fi3 was used to obtain brightfield images from WT, B7 and C12 cells with a 20X objective (S Plan Fluor ELWD 20X/0.45). NIS-elements acquisition software was used to acquire the images with a resolution of 1024 × 1024 pixels.

### Western blot

Cells were briefly washed with PBS, directly lysed in 1X SDS sample buffer and boiled for 5 min. About 20 μg of sample lysates were separated by SDS-PAGE and transferred to nitrocellulose membranes. Fluorescent western blotting was performed as previously described [26]. The following primary antibodies were used: rabbit monoclonal anti-huntingtin (1:500, D7F7, Cat #5656S, Cell Signaling), mouse anti-huntingtin (mEM48 clone, 1:500, Cat #MAB5374, Millipore), mouse monoclonal anti-actin (1:1000, Cat #sc-47778, Santa Cruz Biotechnology). Fluorescent signals were detected with a LI-COR Odyssey Fc system and the images were quantified with the provided Image Studio 2.0 software.

### RNA sequencing and DEGs analysis

RNA was isolated from WT, B7 and C12 HttKO cells using Direct-Zol™ RNA miniprep Plus kit (Cat. #R2071, ZYMO RESEARCH) according to the manufacturer’s instruction. To reduce genomic DNA contamination, a 15 min on-column DNase I treatment at room temperature was performed. RNA samples in triplicates for each group were submitted to Scripps Florida Genomics Core for RNA sequencing and analysis. Purified RNAs were quantified with the Qubit RNA broad-range assay (Thermo Fisher) and the quality of the RNA was assessed using the RNA Nano chip on the Agilent 2100 Bioanalyzer (Agilent Technologies). All RNA had an RNA Integrity Number > 9.2 and was of very high quality. The DNase-treated Total RNA (800 ng) was depleted of ribosomal RNA using species-specific probes provided in the NEBNext Ultra II rRNA-depletion kit Human/Mouse/Rat (Cat. #E6310, New England BioLabs). The rRNA-depleted RNA was processed according to the manufacturer’s protocol to convert to cDNA and final sequence-ready libraries with the NEBNext Ultra II RNA library prep kit (Cat. #E7760, New England BioLabs). The final libraries were sequenced on the NextSeq 500 (Illumina) with paired-end 40 bp reads. On average 30.84, 32.01 and 33.86 million reads were obtained for the WT, B7 and C12 HttKO samples, respectively. The reads had > Q30 base quality scores suggesting less than 1 error in 1000 bp. After quality control and trimming, the reads were mapped to the human genome (ENSEMBL-grch38release 91) using the star version 2.5.2a aligner and gene abundance was estimated with python version 2.7.11, and htseq version 0.11.0. All conditions types were compared in a pairwise manner with all other conditions to test for any genes that were differentially expressed using R version 3.5.1, and deseq2 version 1.22.2.

### Cell proliferation assays

Trypan blue exclusion assay: Cells were seeded in quadruplicate wells of a 96-well plate at 1 × 10^4^ cells per well. Their proliferation was monitored daily for 4 days by trypan blue exclusion method. Cell numbers and viability were determined with a LUNA-II automated cell counter.

Impedance-based real-time cell proliferation measurement: Approximately 1 × 10^4^ cells were seeded in a 16-well electronic microtiter plate (E-plate) (ACEA Biosciences, Inc.). Cells were incubated at room temperature for 30 min and then placed into an xCELLigence Real-Time Cell Analysis (RTCA) instrument inside an incubator. The flow of electric current through the microelectrodes was measured every 15 min over 165 h at 37 °C with 5% CO_2_ supply. The impedance of electron flow caused by adherent cells as an insulator is reported using a unitless parameter called Cell Index (CI), where CI = (impedance at time point n – impedance in the absence of cells)/nominal impedance value.

### Quantitative RT-PCR

RNA samples were prepared the same way as preparing RNA-seq samples as described above. Quantitative RT-PCR was performed using the SYBR green method as we previously described [26, 27]. Primers for each target was selected from the PrimerBank (https://pga.mgh.harvard.edu/primerbank/) [28]. The following primers are: human NTRK2 (NM_006180)-forward: 5′-TCGTGGCATTTCCGAGATTGG-3′, Reverse: 5′-TCGTCAGTTTGTTTCGGGTAAA-3′; human SLC7A2 (NM_001008539)-forward: 5′- CCTTATGGCTTTACGGGAACG-3′, reverse: 5′-CGAGGAGGTAGTACGGCATCA-3′; human GAD1 (NM_000817)-forward: 5′- CCTCAACTATGTCCGCAAGAC-3′, reverse: 5′- TGTGCGAACCCCATACTTCAA-3′; human CHRM2 (NM_000739)-forward: 5′-ACACCCTCTACACTGTGATTGG-3′, reverse: 5′-GTCCGCTTGACTGGGTAGG-3′; human SP1 (NM_001251825)-forward: 5′-TGGCAGCAGTACCAATGGC-3′, reverse: 5′-CCAGGTAGTCCTGTCAGAACTT-3′; human actin (NM_001101)-forward: 5′-CATGGAGTCCTGTGGCATC-3′, reverse:5′-AGCACTGTGTTGGCGTAC-3′.

### Data analysis

All experimental data were expressed as means ± S.D. To establish significance, data were subjected to unpaired student’s *t*-tests or one-way ANOVA followed by the *Tukey's* multiple comparison test using the GraphPad Prism software statistical package 8 (GraphPad Software). The criterion for significance was set at *p* ≤ 0.05.

## Results

### Knocking out Htt expression in SH-SY5Y cells by CRISPR-Cas9

**Fig. 1:**
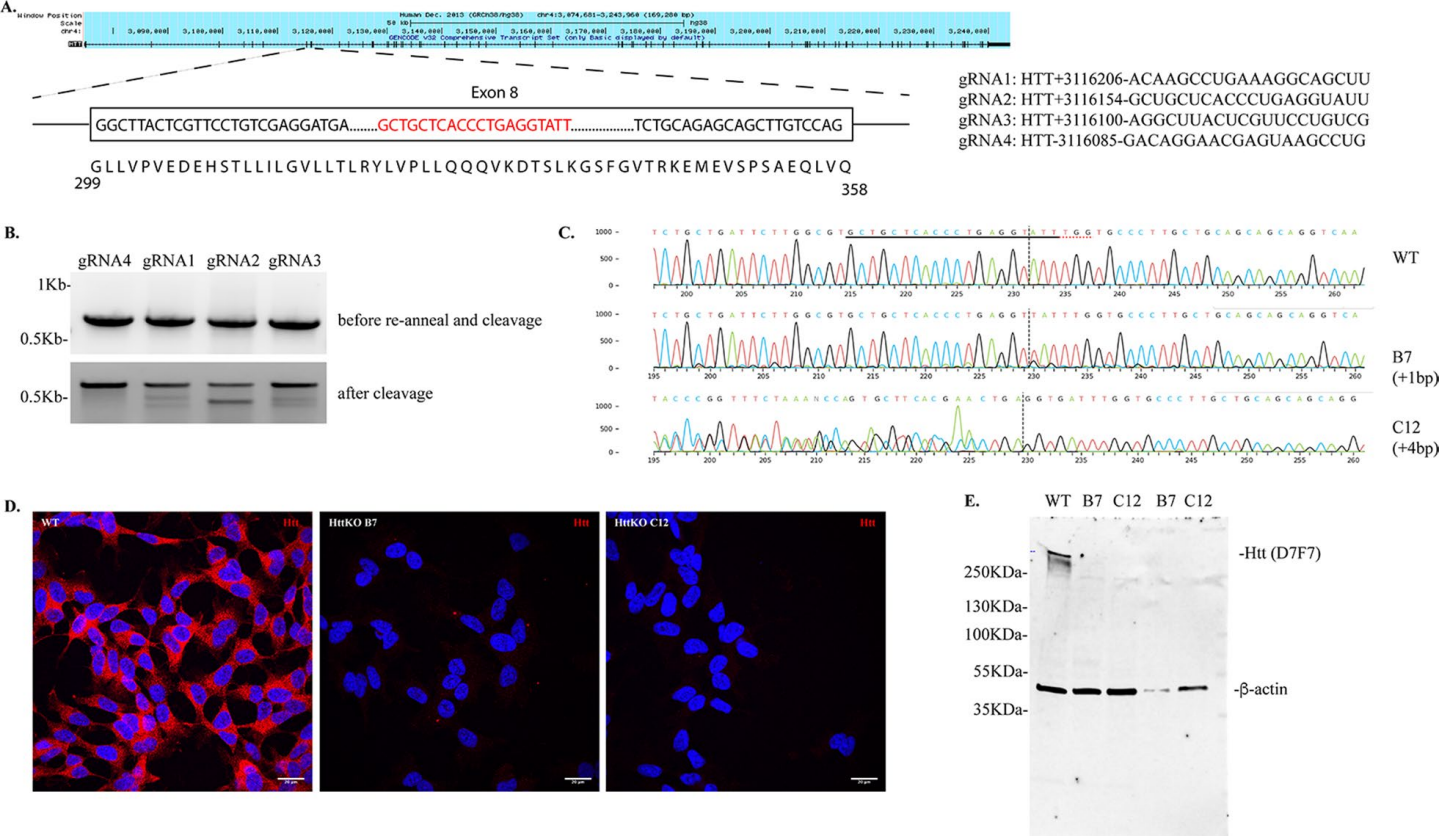
Generation and validation of HttKO clones in SH-SY5Y cells. **A** A diagram depicting the exons of human Htt and four gRNA sequences. The structural map of Htt is generated from the UCSC Genome Browser. Exon 8 is enlarged in the box with the coded amino acid sequence at the bottom. Red sequence indicates the gRNA2 sequence. **B** Estimation of mutation frequencies by mismatch cleavage assay. Forty-eight hours after electroporation, genomic DNA from different gRNA- transfected cells was extracted and the fragment around the gRNA targeted sequence was amplified by PCR, cleaved by mismatch assay and analyzed by DNA gel electrophoresis. Upper panel: PCR products before re-annealing and cleavage. Lower panel: PCR products after mismatch cleavage assay. **C** Sanger sequencing of WT, B7 and C12 clones to identify InDels. **D** Representative confocal images showing the absence of Htt expression in B7 and C12 clones. All images were taken with the same laser intensity and gain. Not further adjustments were applied. Cells were immunostained with rabbit monoclonal Htt antibodies (D7F7). Nuclei were counterstained with DAPI. Scale bar: 20 μm. **E** Western blot analysis of Htt expression in WT SH-SY5Y cells, B7 and C12 HttKO clones. Rabbit monoclonal Htt antibody (D7F7) and mouse monoclonal β-actin antibody were used in the blot

In order to investigate the molecular functions of normal Htt, we first knocked out endogenous Htt in human neuroblastoma SH-SY5Y cells using the CRISPR-Cas9 gene editing approach. Four gRNAs targeting exon 8 of human *HTT* were selected and individually transfected into SH-SY5Y cells by electroporation (Fig. 1A). Mismatch cleavage assays performed 48 h later revealed the highest mutation frequencies in pooled cells transfected with gRNA2 (Fig. 1B, also see Additional file 1: Figure S4 for uncropped image). Therefore, gRNA2-transfected cells were further serial-diluted to obtain single colonies. For initial screening of positive clones, we developed a quick dot blot assay using antibodies against Htt and actin (Additional file 1: Figure S1A). Htt expression levels were normalized to actin for quantification (Additional file 1: Figure S1B). Based on this initial screen, we selected those colonies with less than 50% of Htt expression when compared to that of wild type (WT) SH-SY5Y cells for Sanger sequencing to identify Indel mutations. Out of 17 samples submitted, we totally obtained 5 biallelic clones with different Indel mutations (B7, C12, E2, F3 and H9, analysis of the Sanger sequencing results revealed the presence of < 0.1% of WT sequence) and 2 possible monoallelic HttKO clones (D3 and D5, analysis of the Sanger sequencing results revealed the presence of 45.7% and 55.3% of WT sequence, respectively), which were then confirmed by the absence or reduction of Htt expression in Western blot analysis (Additional file 1: Figure S1C, also see Additional file 1: Figure S4 for uncropped image). Sanger sequencing revealed that the B7 clone had a single insertion of 1 bp and the C12 clone had an insertion of 4 bp (Fig. 1C). Based on these results, we chose clones B7 and C12 for further validation and characterization. We first analyzed off-target effects of gRNA2 at the top predicted off-target sites with three mismatches. We did not detect any mutations by Sanger sequencing using specific primers flanking the selected regions of the potential off-target genes (hLZIC and hChr18, selected by the online Synthego CRISPR design tool). Therefore, B7 and C12 HttKO clones were used in the subsequent studies. Immunostaining with specific antibodies against Htt showed the absence of Htt signaling in B7 and C12 HttKO cells (Fig. 1D). Consistently, Htt expression was not detected in B7 and C12 clones by Western blot using specific Htt rabbit monoclonal antibody (D7F7) which is produced against a synthetic peptide corresponding to residues surrounding Pro1220 of human Htt protein (Fig. 1E, also see Additional file 1: Figure S4 for uncropped image).

### RNA-Seq analysis shows widespread gene dysregulation in the HttKO cells

**Fig. 2:**
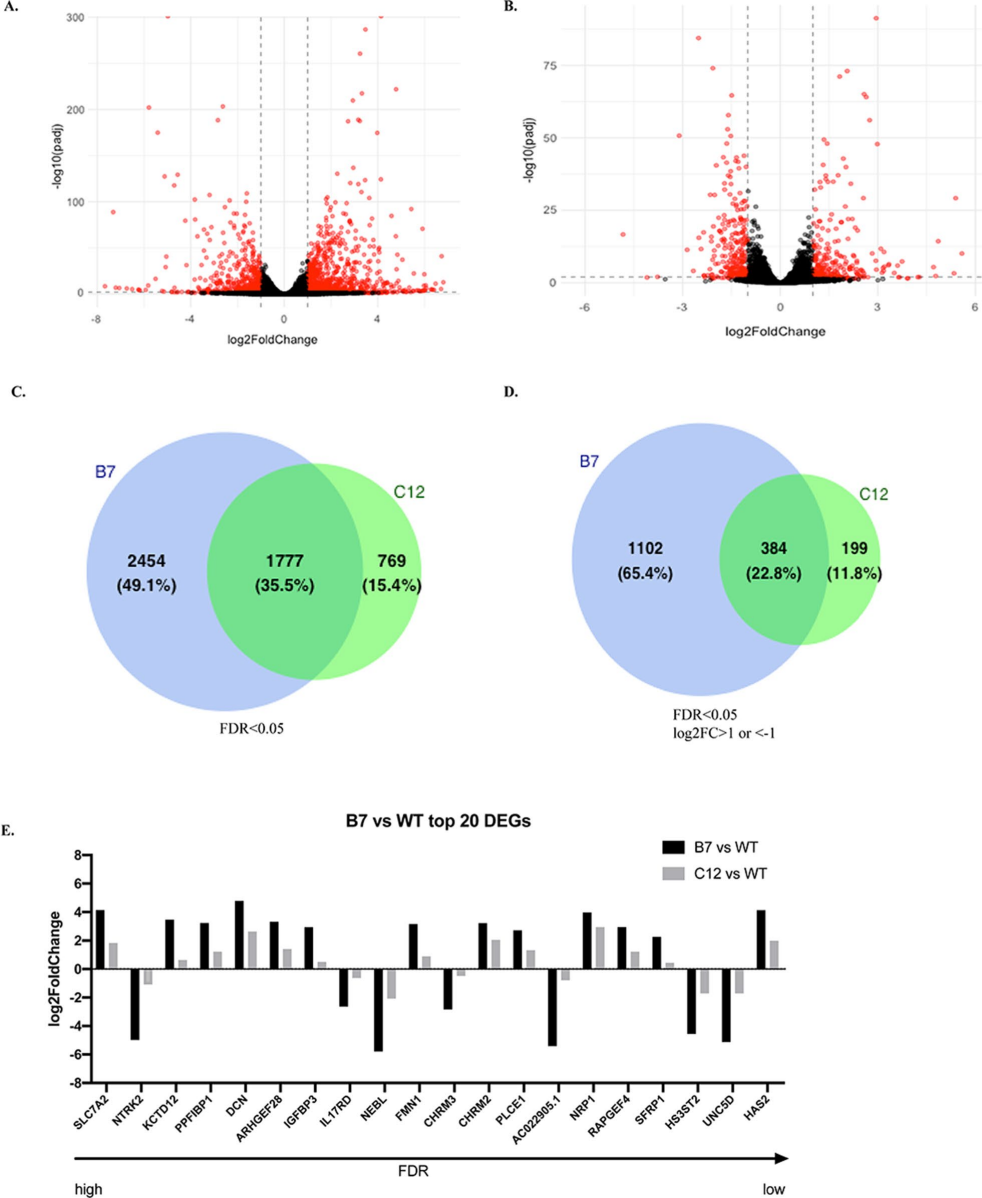
Differential expression analysis of RNA-seq data. **A**, **B** Volcano plot of statistically significant DEGs at FDR < 0.05 for the B7 versus WT group (**A**) and the C12 versus WT group (**B**). The red dots represent DEGs at FDR < 0.01 [i.e., − log10(padj) ≥ 2], with an absolute value of log2FoldChange ≥ 1. **C**, **D** Venn diagrams showing the DEGs identified from the B7 versus WT (B7) and C12 versus WT (C12) groups at FDR < 0.05 (**C**) and FDR < 0.05 with an absolute value of log2FoldChange ≥ 1(D). **E** Top 20 DEGs in the B7 group. The expression levels of the same genes in the C12 group is also included. The horizontal arrow indicates that FDR value decreases from left to right

We started our studies with investigating global transcriptome changes in response to Htt deletion by RNA-seq. Differential expression analysis of pairwise comparison of B7 versus WT and C12 versus WT identified 4231 and 2546 differentially expressed genes (DEGs) with False Discovery Rate (FDR or padj) < 0.05, respectively. The WT, B7 and C12 HttKO cells could be clearly distinguished by the principal component analysis (Additional file 1: Figure S2A). The differences between the B7 and C12 clones could be due to the intrinsic single cellular differences since these two clones were generated from two single cells, respectively. The clear separation of the three genotypes was also evident in the heatmap showing the Euclidian distance between each pair of samples calculated using the rlog transformed data of all genes (Additional file 1: Figure S2B). The volcano plot for the DEGs at FDR < 0.05 are shown in Fig. 2A, B for the B7 versus WT group (indicated as the B7 group hereafter) and the C12 versus WT group (indicated as the C12 group hereafter), respectively. We then applied different thresholds to narrow down the DEGs for further analysis (Additional file 1: Table S1). In general, the C12 group has less DEGs identified when compared to the B7 group. Additionally, more up-regulated DEGs were identified than down-regulated DEGs (Additional file 1: Table S1). We found a large overlap in DEGs between B7 and C12 groups as shown by Venn Diagram at two thresholds, FDR < 0.05 (Fig. 2C) and FDR < 0.05 with an absolute value of log2FoldChange ≥  1(Fig. 2D).

Finally, the top 20 most significant DEGs based on FDR values were selected from the B7 group and their fold changes were plotted for both the B7 and C12 groups (Fig. 2E). All DEGs, despite of showing different fold of changes, in both groups share similar trends of up- or down-regulation. To demonstrate the consistency of DEG levels among individual samples, the heat maps of the top 50 DEGs in individual samples of B7 and C12 groups are shown in the Additional file 1: Figure S3.

Taken together, while B7 and C12 HttKO cells are generated from two different single cells which could explain the differences among DEGs in the RNA-seq analysis, they share high similarities in the regulation patterns due to the knockout of Htt expression. We therefore proceeded with the common DEGs (230 up-regulated and 145 down-regulated) between B7 and C12 at FDR < 0.05 with an absolute value of log2FoldChange ≥ 1(Fig. 2D) for further functional analysis. The list of common DEGs is provided in the Additional file 2: File 1.

### Functional analysis of DEGs using gene ontology (GO) analysis

**Fig. 3:**
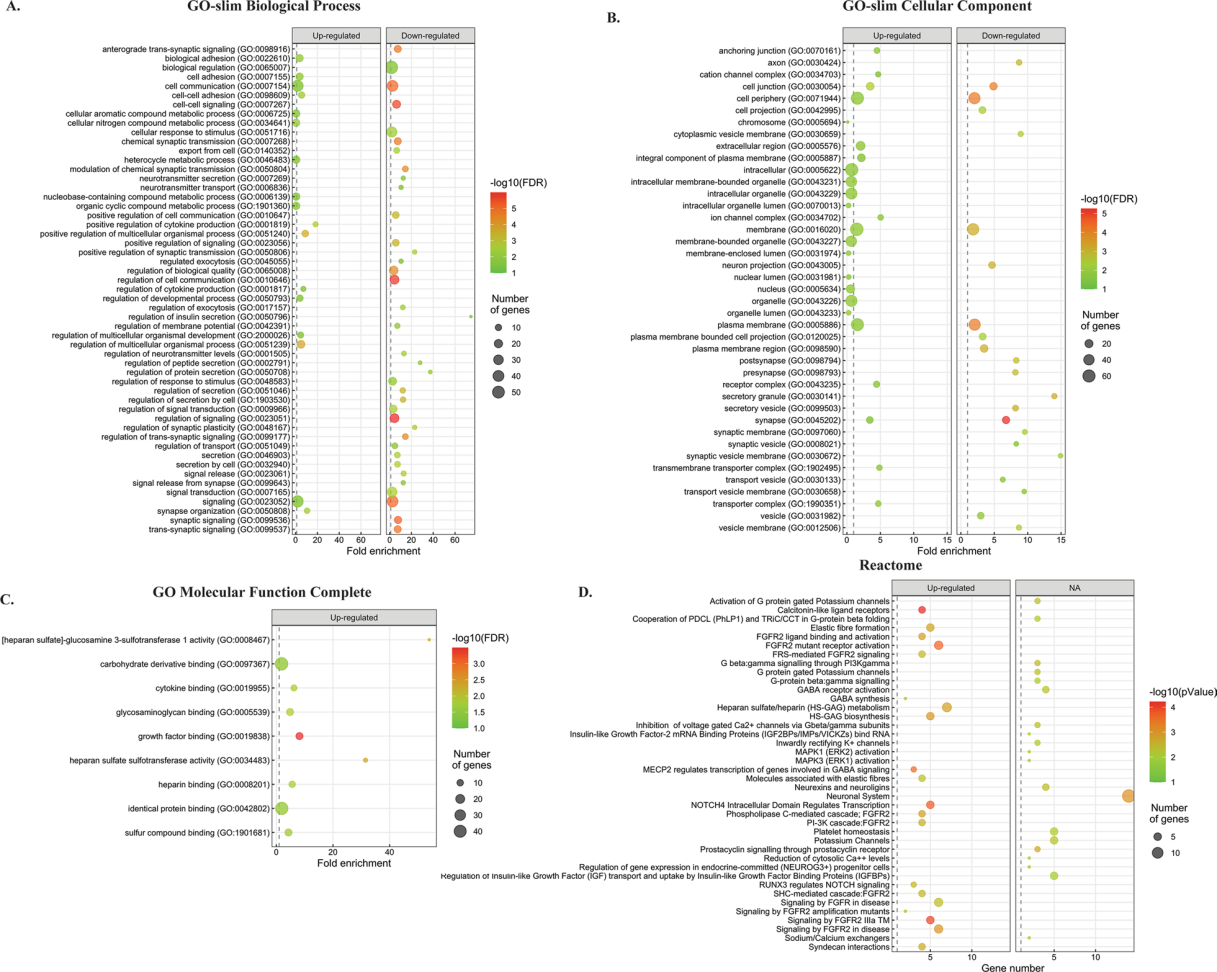
Go and Reactome enrichment pathway analysis of up- and down-regulated DEGs. **A** Dot plot shows GO terms of Biological Process identified by Panther GO-slim analysis. **B** Dot plot shows GO terms of Cellular Component identified by Panther GO-slim analysis. **C** Dot plot shows significantly enriched GO terms based on molecular function (FDR < 0.05) of up-regulated DEGs identified by Panther GO complete analysis. **D** Dot plot shows top 20 enriched Reactome pathways. The size of the dot is based on gene count enriched in the pathway, and the color of the dot shows the pathway enrichment significance

In order to investigate the functional associations of the common DEGs, we performed GO analysis using Gene Ontology (http://geneontology.org) and PANTHER (Protein Analysis Through Evolutionary Relationships, http://pantherdb.org). We first used PANTHER GO-Slim analysis which has manually curated annotations [29]. If GO-Slim analysis did not return significant terms, we then used GO complete analysis as indicated. GO-slim analysis based on Biological Process (BP) revealed 17 enriched BP pathways from up-regulated DEGs and 38 enriched BP pathways from down-regulated DEGs with an FDR < 0.05. Figure 3A shows all the BP pathways. Clearly, both up- and down-regulated genes are highly enriched for terms associated with cell communication (GO:0007154) and signaling (GO:0023052). Interestingly, biological adhesion process, multicellular organism process and metabolic process are more enriched in the up-regulated DEGs (Fig. 3A), while processes related to synaptic signaling are more accumulated in the down-regulated DEGs, indicating that loss of Htt may preferentially impair synaptic functions (Fig. 3A). The affected systems include neurotransmitter receptors related to acetylcholine, glutamate, GABA and dopamine.

Go-Slim analysis based on Cellular Component revealed an enrichment of intracellular membrane-bound components in the up-regulated genes (24 pathways in total with an FDR < 0.05) and synaptic components in the down-regulated genes (22 pathways in total with an FDR < 0.05) (Fig. 3B), which is consistent with the GO analysis of biological processes. Molecular Function based on GO complete analysis revealed a significant enrichment of growth factor binding in the up-regulated genes (Fig. 3C) and did not yield any functions significantly enriched in the down-regulated genes.

### Reactome pathway analysis

We next analyzed the biological pathways using Reactome overrepresentation analysis (http://Reactome.org) [30, 31]. In general, 133 out of 230 up-regulated DEGs were found in Reactome, where 532 pathways were hit by at least one of them. 73 out of 145 down-regulated DEGs were found in Reactome, where 458 pathways were hit by at least one of them. The top 20 most relevant pathways sorted by p-Value are shown in Fig. 3D for both up- and down-regulated DEGs, respectively. Consistent with the GO term analysis, many up-regulated genes are involved in growth factor binding and signaling (Fig. 3D) and neuronal system related signaling pathways are the top pathways enriched in the down-regulated DEGs (Fig. 3D). The full list of identified Reactome pathways is listed in the Additional file 2: File 1.

### Transcription factors (TFs) in regulating DEGs

Htt can regulate gene transcription by mediating bindings between TFs and transcriptional regulators [19]. We therefore searched for TFs that may be involved in regulating the expression of DEGs using TRRUST V2, an expanded reference database for human and mouse transcriptional regulatory interactions [32]. All candidate TFs identified in regulating DEGs are listed in Table 1. REST (RE1-silencing transcription factor) is significantly associated with transcriptionally regulating DEGs. SP1, although less significant, regulates 11 DEGs from the submitted list. The expression level of most of the TFs listed in Table 1 was only slightly increased or decreased in HttKO cells from both B7 and C12 cell lines. Specifically, SP1 was slightly increased by 16% and 10% in B7 and C12 HttKO cells respectively, (B7: Log2FC = 0.21, *p* = 0.0465; C12: Log2FC = 0.17, *p* = 0.14) and REST was significantly increased by 50% and 37% in B7 and C12 HttKO cells respectively (B7: Log2FC = 0.62, *p* = 1.84E-11; C12: Log2FC = 0.45, *p* = 7.45E-6).

**Table 1:** Candidate key TFs for up- and down-regulated DEGs

#	Key TF	Description	# Of overlapped genes	*P* value	FDR
1	REST	RE1-silencing transcription factor	6	1.39e−05	0.000361
2	MYB	v-myb myeloblastosis viral oncogene homolog (avian)	4	0.00341	0.0443
3	POU5F1	POU class 5 homeobox 1	3	0.00744	0.0485
4	FOXF2	Forkhead box F2	2	0.00746	0.0485
5	HOXB7	Homeobox B7	2	0.0117	0.061
6	KLF5	Kruppel-like factor 5 (intestinal)	2	0.0197	0.0852
7	FOXO1	Forkhead box O1	2	0.0616	0.185
8	EGR1	Early growth response 1	4	0.062	0.185
9	PARP1	Poly (ADP-ribose) polymerase 1	2	0.0662	0.185
10	RARA	Retinoic acid receptor, alpha	2	0.071	0.185
11	RUNX3	Runt-related transcription factor 3	2	0.091	0.208
12	DNMT1	DNA (cytosine-5-)-methyltransferase 1	2	0.0962	0.208
13	JUN	Jun proto-oncogene	5	0.109	0.218
14	PPARA	Peroxisome proliferator-activated receptor alpha	2	0.141	0.247
15	NFKB1	Nuclear factor of kappa light polypeptide gene enhancer in B-cells 1	8	0.143	0.247
16	HIF1A	Hypoxia inducible factor 1, alpha subunit (basic helix-loop-helix transcription factor)	3	0.166	0.269
17	SP1	Sp1 transcription factor	11	0.177	0.271
18	CEBPA	CCAAT/enhancer binding protein (C/EBP), alpha	2	0.213	0.308
19	WT1	Wilms tumor 1	2	0.251	0.343
20	SPI1	Spleen focus forming virus (SFFV) proviral integration oncogene spi1	2	0.282	0.367
21	USF1	Upstream transcription factor 1	2	0.301	0.373
22	RELA	v-rel reticuloendotheliosis viral oncogene homolog A (avian)	6	0.399	0.471
23	STAT1	Signal transducer and activator of transcription 1, 91 kDa	2	0.417	0.471
24	CREB1	cAMP responsive element binding protein 1	2	0.451	0.475
25	YY1	YY1 transcription factor	2	0.457	0.475
26	TP53	Tumor protein p53	3	0.526	0.526

### Knocking out Htt affects cell proliferation

**Fig. 4:**
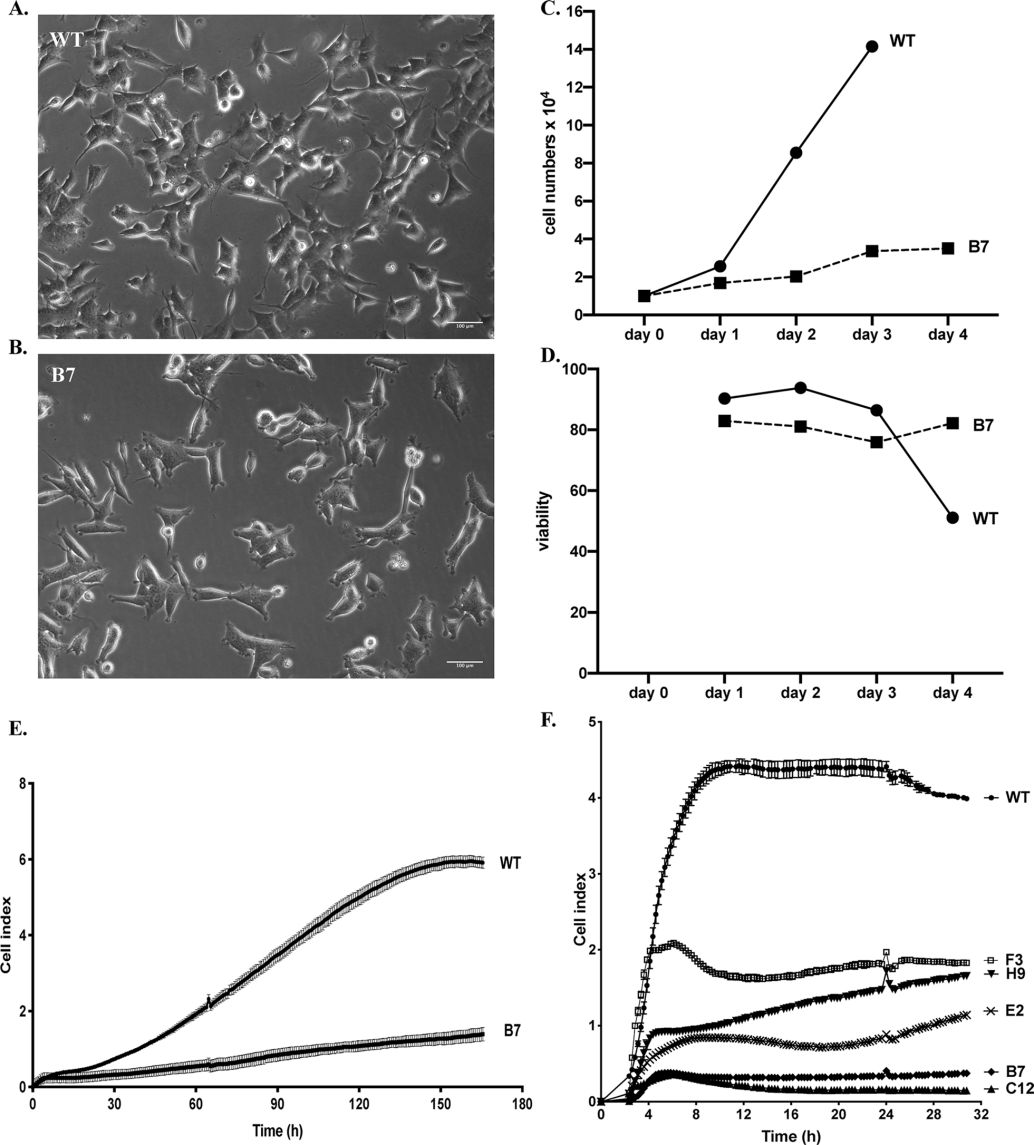
Cell proliferation was reduced in B7 HttKO cells. **A**, **B** Representative light microscopic images showing the cell morphology of WT (**A**), B7 (**B**) and C12 (**C**) cells. scale bar: 100 μm. **C** Cell proliferation rate was determined by daily cell counting for up to 4 days using the trypan blue exclusion method. **D** Cell viability measurement for up to 4 days using the trypan blue exclusion method. **E** Real-time monitoring cell proliferation by impedance measurement. Cells were seeded at 1 × 10^4^ cells per well. Cell adherence, spreading and proliferation were monitored every 15 min for 165 h using the xCELLigence real time cell analysis system. N = 4 for each cell type. **F** Real-time monitoring cell proliferation by impedance measurement. Cells were seeded at 3 × 10^4^ cells per well. Cell adherence, spreading and proliferation were monitored every 15 min for 30 h using the xCELLigence real time cell analysis system. N = 2 for each cell type

We noticed that HttKO cells took longer time to reach to confluency compared to WT SH-SY5Y cells during culture. We did not observe a significant difference in morphology under a light microscope, except that more rounded cells were present in HttKO cells, indicating cell death (Fig. 4A, B). We then analyzed cell proliferation in WT and B7 HttKO cells by counting cell numbers daily for up to 4 days. Consistent with our observation, cell proliferation was markedly reduced in the B7 HttKO cells as demonstrated by a slower daily increase in cell numbers (Fig. 4C). Although the cell viability was lower in B7 HttKO cells compared to WT, we did not detect a significant decrease of viability in B7 HttKO cells over the 4 days. The viability of WT cells was significantly decreased at day 4 due to the over-confluency observed under the microscope (Fig. 4D). Therefore, cell number counting for WT cells at day 4 was not included (Fig. 4C). These data indicate that the reduced increase in cell number over the 4 days in B7 HttKO cells is largely due to the impaired proliferation rather than increased cell death. We then monitored cell proliferation in real time using the noninvasive electrical impedance measurement. Adherent cells act as an insulator on the surface of the electrode and change the ionic medium of the electrode solution, thus increasing the impedance. Expectedly, increased impedance is directly related to increased number of cells present on the electrode surface before cells reach to confluency [33]. Cellular impedance is converted into cell index, which directly correlates to impedance. A steady increase of cell index therefore reflects cell proliferation until cells became confluent. Cell index was constantly lower in B7 HttKO cells compared to WT cells at all time points examined, indicating cell proliferation was slower in B7 HttKO cell lines (Fig. 4E). Using this automatic, high-throughput impedance assay, we further demonstrated that other HttKO clones had decreased cell proliferation (Fig. 4F). Taken together, these data suggest that knocking out Htt expression slowed down cell proliferation. Decreased cell growth and proliferation was also observed in Hdh-null embryonic stem cells and neurons [34]., which is in line with the affected biological processes revealed from the RNA-seq analysis. We confirmed this essential role for huntingtin on RNA-seq analysis, detecting the general dysregulation of genes involved in cell development, differentiation and neurogenesis (Fig. 3A).

### RNA-seq data validation

**Fig. 5:**
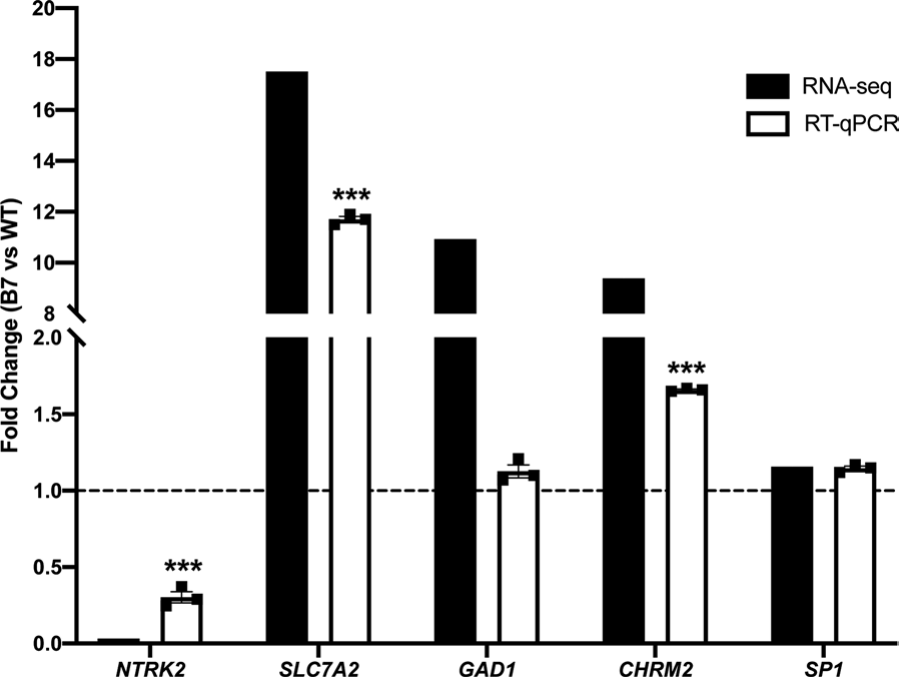
RT-qPCR analysis of several DEGs. N = 3/group. ***p < 0.001 compared to the WT group, by students’ t-test

Seven DEGs were evaluated for their expression patterns in WT and B7 HttKO cells by RT-qPCR to validate the RNA-Seq data. Consistent with the RNA-seq data, NTRK2 mRNA levels, measured by RT-qPCR, were significantly reduced in B7 HttKO cells (Fig. 5). Similarly, the levels of SLC7A2 mRNA, CHRM2 mRNA were significantly increased in B7 HttKO cells (Fig. 5). SP1 level did not change in both RT-qPCR and RNA-seq data. Although GAD1 levels were significantly increased in RNA-seq data, we only detected a 23% increase in the RT-qPCR analysis (Fig. 5). This could be due to the different sensitivities of these two methods. In general, the qRT-PCR results validated the expression pattern of selected DEGs from the RNA-Seq data.

## Discussion

In this study, we generated SH-SY5Y HttKO cells using the CRISPR/Cas9 genetic editing approach in order to better understand the function of normal Htt. The main effort here was to analyze the effect of Htt deletion on gene expression. Although SH-SY5Y cells have neuroblast-like morphology [35], this transcriptome analysis provides important clues to further investigate the molecular function of normal Htt during neuron development and in mature neurons.

By performing RNA-seq analysis on two independent HttKO clones, we have identified a large number of up-regulated and down-regulated genes, indicating that knocking out huntingtin has widespread effects at the transcriptome level. In the present study, we performed GO and Reactome enrichment analysis of the DEGs and showed that genes involved in pathways related to multicellular organism development, neuron development, neurotransmission, extracellular matrix processing and organization, and nucleosome dynamics are dysregulated. We further examined the transcription factors that may regulate these DEGs. Consistent with the disrupted pathways associated with cellular development, we showed that Htt-null cells exhibited reduced cell proliferation than wild type cells.

GO analysis of DEGs based on Biological Process indicates that both up-regulated and down-regulated DEGs are associated with multicellular organism development and processes, especially with neuron development. This is consistent with the essential role of normal Htt in early embryogenesis and CNS development [3–5, 8]. In patients with HD, neural development is also altered through disrupted neurogenesis [36]. Pathways or genes identified from our study can provide further clues to investigate the molecular action of muHtt in neuron development. We also observed decreased cell proliferation in HttKO cells. The underlying molecular mechanism remains unclear. It was suggested that normal Htt functions to counterbalance apoptosis as high incidence of apoptosis was observed in *Hdh* null embryos [3]. Knocking out Htt affects mitotic spindle orientation by mislocalizing the dynein/dynactin complexes and therefore alters cell fate of cortical progenitors of the ventricular zone in mouse embryos [37]. Our data further suggest that deleting Htt causes widespread transcriptome changes that accompany alterations in multiple signaling pathways important during development. Of particular interest, the transcription of genes involved in ECM production and organization were significantly changed in response to knocking out Htt (Additional file 2: File 1 and Fig. 3). Specifically, we identified a significant increase in genes producing metalloproteinases, which are important for cell migration, proliferation and apoptosis [38, 39] and play important roles in the CNS development [40, 41]. It has been shown that Htt regulates neurulation and rosette formation by inhibiting ADAM10 activity and N-cadherin cleavage [42]. Furthermore, defects in neural tube morphogenesis in Htt knockdown zebrafish embryos could be rescued after treatment with an ADAM10 inhibitor [42], indicating that knocking down Htt expression increased ADAM10 activity. ADAM10 is pathologically active in HD [43]. The level of ADAM10 mRNA was slightly increased in our RNA-seq analysis (Log2FoldChange = 0.277, FDR = 0.051). The role of other MMPs or ADAMs identified in our study in Htt-mediated pathways need to be further investigated. In support of the role of metalloproteinases in HD pathogenesis, altered MMP mRNA levels, including MMP-2, -9, -10, -11, -14, -15, -16, -17, -28, and activities have been reported in different HD models [44, 45].

Our RNA-seq analysis detected significant changes in pathways related to synaptic signaling and transmission in response to Htt deletion. Neurotransmitter systems including acetylecholine, glutamate, dopamine and GABA appear to be mostly affected. CHRM2, a muscarinic acetylcholine receptor subtype 2, was were up-regulated and CHRNB2, a nicotinic receptor beta 2 subunit, was down-regulated. Additionally, metabotropic glutamate receptor (GRM1), GABA type A receptor subunits (GABRB3) and dopamine D2 receptor (DRD2) were significantly down-regulated. The transcripts of GAD1, the enzymes responsible for synthesizing GABA, was increased in HttKO cells. The net functional outcomes of these disruptions in neurotransmission remains to be experimentally investigated at the cellular and behavioral levels. In contrast, CHAT expression was decreased in YAC72 [13] and R6/1 HD mouse brains [46]. Cholinergic transmission is affected in R6/1 mice, resulting in severe deficits in learning and reference memory [46]. GAD1 mRNA was also decreased in R6/2 mouse brains [47] or neurons differentiated from HD induced pluripotent stem cells [48]. Restoring GABAergic transmission rescues memory deficits in the R6/2 mouse model [49]. The studies on the effect of knocking out/down normal Htt in adult mice have been mainly focused on assessing motor functions [9–11]. To fully understand the consequences of therapeutically knocking out/down Htt in the adult CNS, our data point out the importance of performing multiplex behavioral assessments, including cognition, mood regulation and motor functions.

Transcriptional regulation is one of well-known functions of normal Htt. We here identified a list of genes whose expression could be directly or indirectly regulated by Htt. A number of studies indicate that Htt regulates gene transcription by binding to a number of TFs, including, but are not limited to, p53 and CREB-binding protein (CBP) [50], NeuroD [51], methyl-CpG binding protein 2 (MeCP2) [52], REST[13], SP1 and its co-activator TAFII130 [53]. Most of these TFs mRNAs were not significantly changed in HttKO cells from our RNA-seq analysis. REST is well-studied transcriptional regulator that interacts with Htt. REST is considered as a master transcriptional regulator of neuron-specific genes and dysregulation of REST has been implicated in a number of neurodegenerative diseases [54, 55]. Cytosolic normal Htt interacts with REST and reduces its availability to the nuclear repressor element 1/neuron restrictive silencer element (RE1/NRSE) binding site which allows gene transcription [13]. Mutant Htt has reduced affinity for REST, allowing more REST to enter the nucleus and inhibit transcription of genes, such as BDNF, a key neurotrophic factor that is reduced in HD [13, 56, 57] and other NRSE-containing genes [13]. SP1 is a ubiquitously expressed, zinc finger-containing DNA binding protein that can activate or repress transcription in response to physiological and pathological stimuli [58]. The binding site of SP1 has been known to be a GC box, with the consensus sequence 5′-(G/T)GGGCGG(G/A)(G/A)(C/T)-3′ [59, 60]. Mutant Htt has a higher affinity for SP1 and its co-activator TAFII130 than normal Htt, leading to a decreased binding of SP1 to DNA [53]. Using a consensus SP1 binding site as a probe, the same study found a 70% decrease in Sp1 binding to DNA in the presence of mutant Htt and a 20% decrease in the presence of wild-type huntingtin [53]. It is conceivable that knocking out Htt frees up more SP1 to regulate genes with SP1-binding sites in their promoters. Clearly, SP1 and REST activities were mainly studied in the context of HD. However, how normal huntingtin control their activity at physiological levels remains elusive. Our report provides the direct evidence that knocking out Htt can affect TF activities, possibly through the loss of Htt-TF interactions. In the absence of Htt, we would expect more REST to be present in the nucleus to inhibit transcription of certain genes. This is the case in our study for several down-regulated DEGs targets for REST (Table 1). However, due to the complexity of TF regulatory networks, it is difficult to determine the mechanism underlying the transcriptional changes of DEGs based on the action of a single TF.

Additionally, it is possible that Htt may directly regulate gene transcription. Although Htt is not recognized as a typical TF due to the lack of a DNA consensus binding sequence, it was reported that Htt can directly bind to DNA promoters and alter DNA conformation and TF binding activity in a polyQ-dependent manner [14]. Chromatin immunoprecipitation (ChIP) combined with DNA array identified the genomic binding sites of normal and mutant Htt in immortal striatal HD cell lines, STHdhQ7 and STHdhQ111 cells, respectively. Interestingly, a survey of the identified gene list from the study (Supplementary file 1 from reference 14) shows that normal Htt, but not mutant Htt binds to the *GAD1* promoter. It is possible that the absence of Htt alters the conformations of some genes, thus allowing the access of different TFs to control their transcription.

## Conclusions

In summary, our RNA-seq analysis has demonstrated a widespread transcriptional change resulting from Htt deletion in human SH-SY5Y cells. It could be directly caused by the loss of Htt-mediated transcriptional regulation or due to the secondary consequences of disruption in the gene regulatory network. This unbiased approach uncovers novel molecular targets to further study the molecular pathways mediated by normal and mutant Htt. On the other hand, Htt is a scaffold protein and interacts with a number of proteins to carry out its functions in trafficking, autophagy and other cellular processes [19, 61, 62]. A comprehensive understanding of Htt molecular functions, therefore, should emerge from interactive studies at the genomic and proteomic levels that aim at identifying direct gene targets and Htt-interacting proteins.

## Supplementary Information

**Additional file 1.**  Supplementary Figures and Table.

**Additional file 2.** Supplementary Excel File containing: 1.List of differentially expressed DEGs present in both B7 and C12 HttKO cells compared to WT cells with a cutoff of FDR<0.05 and the absolute value of log2FoldChange >1 (Table 1). 2.A full list of Reactome pathways associated with the up- and down-regulated DEGs (Tables 2 and 3).

## Data Availability

The authors declare that the data supporting the findings of this study are available within the article and its supplementary information files. The datasets generated and/or analyzed during the current study are available in the GEO repository (GSE178467).

## Abbreviations

CRISPR: Clustered regularly interspaced short palindromic repeats
Cas9: CRISPR-associated protein 9
DEGs: Differentially expressed genes
FDR: False discovery rate
GO: Gene ontology
gRNA: Guidance RNA
Htt: Huntingtin
KO: Knockout
REST: RE1-silencing transcription factor
RE1/NRSE: Repressor element 1/neuron restrictive silencer element
SP1: Sp1 transcription factor
TF: Transcription factor
WT: Wild type
