# Supplementary material for: RNA-seq analysis reveals significant transcriptome changes in huntingtin-null human neuroblastoma cells
Source: BMC Med Genomics. 2021 Jul 2;14:176. doi: 10.1186/s12920-021-01022-w (PMC8252266; doi:10.1186/s12920-021-01022-w)
Supplement: Supplementary file 1 — Additional file 1. Supplementary Figures and Table. [file 12920_2021_1022_MOESM1_ESM.docx]

**Supplementary Tables**

**Table S1:**

| **Table 1. Number of DEGs filtered at different threshold** | | |
| --- | --- | --- |
| Filter threshold | Total DEGs  (up/down) in B7 *vs.* WT | Total DEGs  (up/down) in C12 *vs.* WT |
| FDR<0.05 | 4231  (2142/2089) | 2546  (1072/1474) |
| FDR<0.05  *\|Log2FC\|>1 | 1486  (939/547) | 583  (329/254) |
| FDR<0.01  *\|Log2FC\|>1 | 1275  (803/472) | 457  (223/234) |
| FDR<0.01  *\|Log2FC\|>2 | 478  (332/146) | 82  (58/24) |
| \|log2FC\|: absolute value of log2FoldChange | | |

**Legends to Supplementary File 1**

The following information is included in the Supplementary File 1:

1. List of differentially expressed DEGs present in both B7 and C12 HttKO cells compared to WT cells with a cutoff of FDR<0.05 and the absolute value of log2FoldChange >1 (Tab 1).
2. A full list of Reactome pathways associated with the up- and down-regulated DEGs (Tab 2 and 3).

**Legends to Supplementary Figures**

**Fig. S1.** **Screening of HttKO clones in SH-SY5Y cells.** **A.** A high-throughput dot blot assay to screen single clones for knocking out Htt expression. The right panel shows the layout of the dot blot. Cells from each single clone were lysed in RIPA buffer (50mM Tris-HCl, pH=8.0, 150mM NaCl, 1% NP-40, 0.5% sodium deoxycholate, 0.1% SDS) supplemented with 1% protease inhibitor cocktail (Sigma, St. Louis, MO) at 4°C for 30 minutes. Lysates were cleared by centrifugation for 15 minutes at 16,000 x *g* at 4°C. Supernatants were mixed with 2% β-mercaptoethanol and heated at 70-80° C for approximately 10 minutes. One microliter of each heat denatured sample in triplicates was blotted onto a nitrocellulose membrane and allowed to dry for approximately 20 minutes. Membrane was then proceeded as Western blot. Primary antibodies against Htt (D7F7, 1:500) and β-Actin (C4, 1:1000, sc-47778) were used. Buffer alone was used as the negative control. HEK293 cells transfected with full length Htt23Q and Htt145Q were used as positive controls. **B.** The intensity of each dot was quantified and Htt expression was normalized with actin expression. The red horizontal line indicates the expression threshold of 20% of Htt expression in WT cells. The green horizontal line indicates the expression threshold of 50% of Htt expression in WT cells. Red arrows indicate the positive clones later confirmed by Sanger sequencing. **C.** Western blot analysis of Htt expression in WT SH-SY5Y cells and different HttKO clones. The lysate prepared from SH-SY5Y cells transfected with full length Htt23Q was used as a positive control. Rabbit monoclonal Htt antibody (D7F7) and mouse monoclonal β-actin antibody were used in the blot.

**Fig. S2. Sample variation analysis. A.** Principle component analysis of all samples. Triplicate samples were submitted for each cell type. **B.** Heatmap of the sample to sample distances. The Euclidean distance between each pair of samples was calculated using the rlog transformed data of all genes. The scale is the Euclidian distance between two samples in multi-dimensional space which is also represented in different shades of blue. Each sample is most closely related to itself (with a Euclidian distance of 0) which explains the perfect relatedness (dark blue) along the diagonal.

**Fig. S3. Heatmap of the top 50 most significant DEGs in the B7 (A) and C12 (B) group.** Values are the rlog transformed raw counts.

# Fig. S4. ﻿Uncropped images for all DNA gels and western blots. Uncropped images of DNA gels/western blots obtained from the LI-COR imaging system are provided. The boxes denote cropped images that are presented in the manuscript (Fig. 1B, Fig. 1E and supplementary Fig. 1C).

**Supplementary Figure S1.**


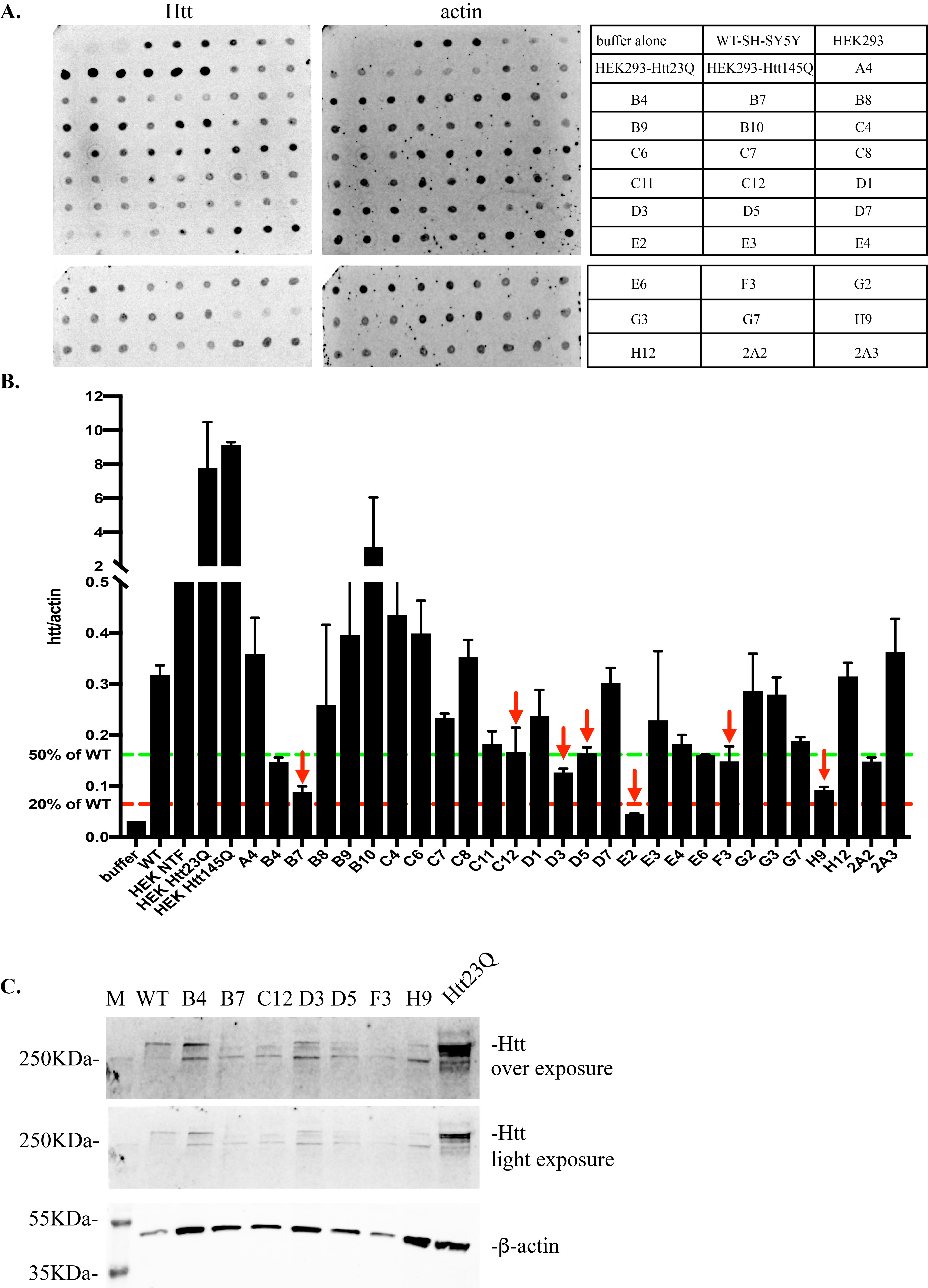


**Supplementary Figure S2.**


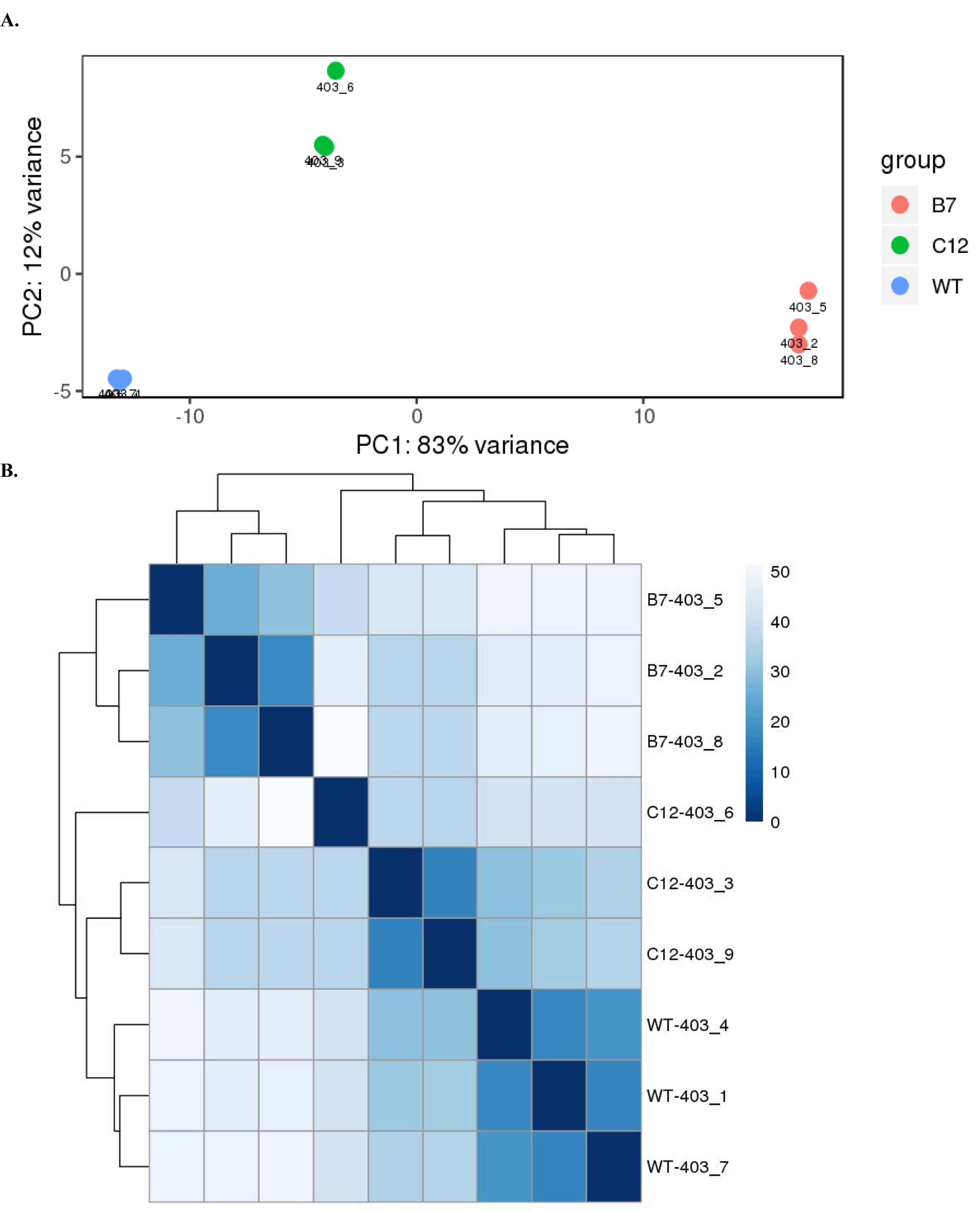


**Supplementary Figure S3.**

**
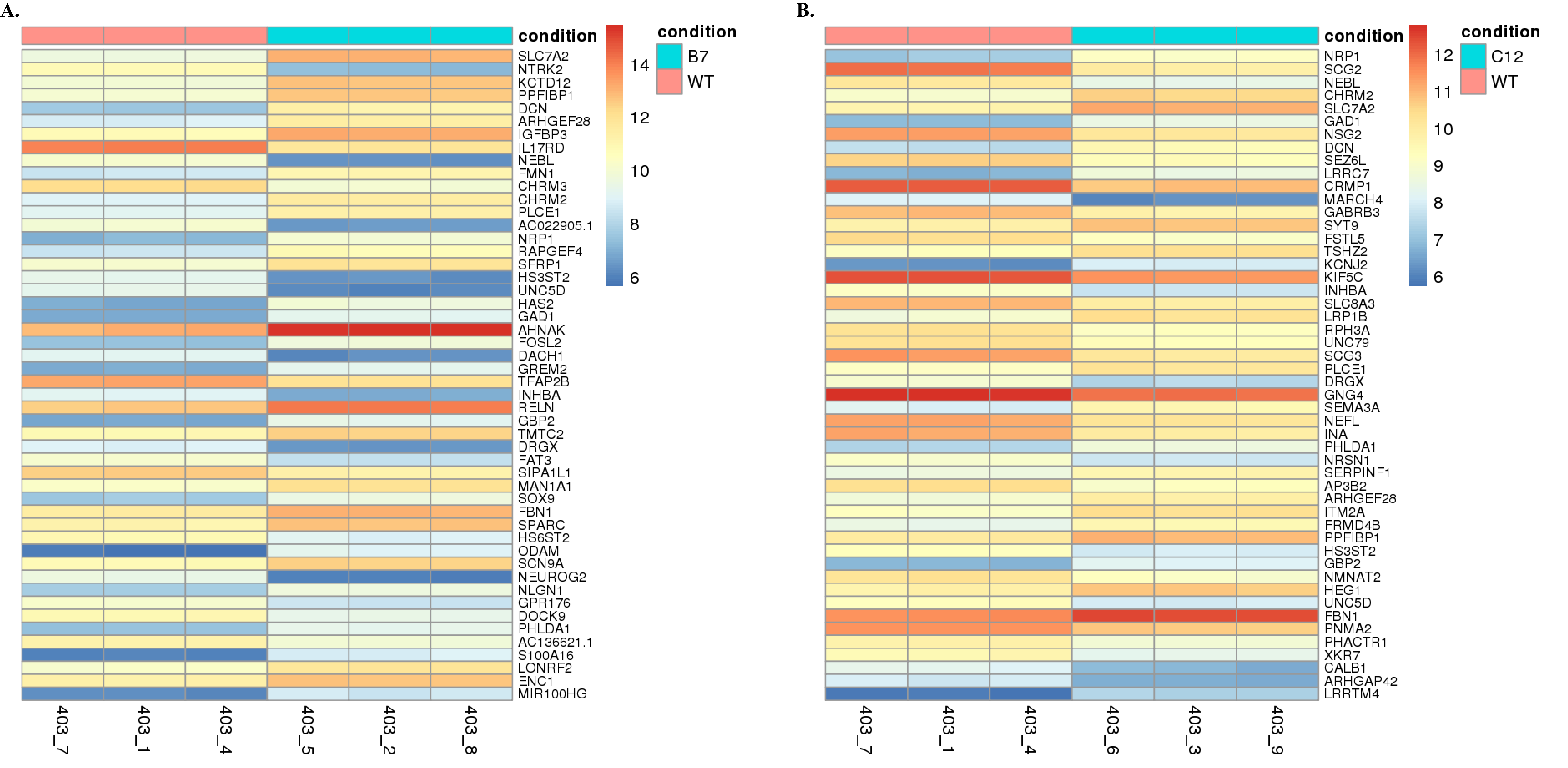
**

**Supplementary Figure S4.**

**
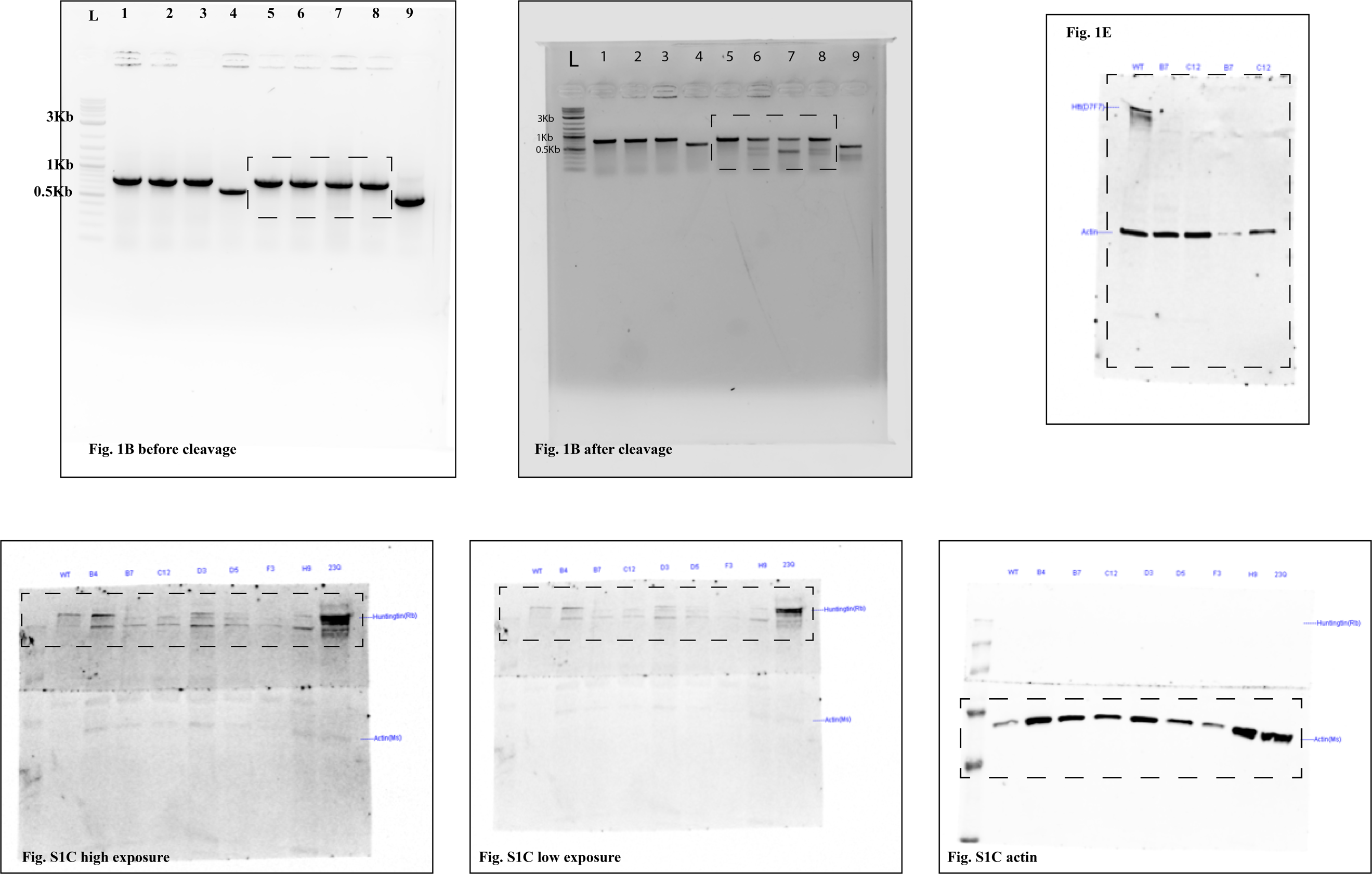
**

In Fig. 1B uncropped images: 1. Cells transfected with pcDNA-mCherry alone; 2. Cells in electroporation buffer alone; 3. Cells transfected with cas9 alone; 4. Cells transfected with RELA gRNA+cas9 as positive control; 5. Cells transfected with Htt gRNA4+cas9; 6. Cells transfected with Htt gRNA1+cas9; 7. Cells transfected with Htt gRNA2+cas9; 8. Cells transfected with Htt gRNA3+cas9; 9. Positive control of mismatch assay provided by the kit.
